# Immune Suppression, Preexisting Immunity, and Mutation Tendency Shaped SARS-CoV-2 Evolution in Persistent Infection

**DOI:** 10.3390/microorganisms13112613

**Published:** 2025-11-17

**Authors:** Minghui An, Xiaolong Dong, Yang Gao, Jinming Ouyang, Haibo Ding, Zheming Zhu, Linan Bao, Yonghui Feng, Wen Tian, Pan Wang, Xiaoxu Han, Hong Shang

**Affiliations:** 1 State Key Laboratory for Diagnosis and Treatment of Infectious Diseases, NHC Key Laboratory of AIDS Prevention and Treatment, National Clinical Research Center for Laboratory Medicine, The First Hospital of China Medical University, China Medical University, Shenyang 110001, China; michelamh@hotmail.com (M.A.); ding_haibo@126.com (H.D.); tianwen_cmu@126.com (W.T.); pan_wang01@163.com (P.W.); 2 Key Laboratory of AIDS Immunology, Chinese Academy of Medical Sciences, Shenyang 110001, China; 3 National Clinical Research Center for Laboratory Medicine, Department of Laboratory Medicine, The First Hospital of China Medical University, Shenyang 110001, China; longer32@163.com (X.D.); gaoyang2417@163.com (Y.G.); ouyang527@hotmail.com (J.O.); zzm466680382@icloud.com (Z.Z.); baolinan364227@163.com (L.B.); yhfeng@cmu.edu.cn (Y.F.)

**Keywords:** SARS-CoV-2, persistent infection, immunocompromised host, evolution

## Abstract

SARS-CoV-2 evolution in persistent infection, which may induce long COVID-19, is predominantly manifested in immunocompromised hosts, who act as the viral reservoirs for future outbreaks. Therefore, understanding the evolutionary mechanisms of novel variants that can evade preexisting immune responses is critical to guide public health measures and develop vaccines tailored for vulnerable populations. We used next-generation sequencing and phylogenetic methods to delineate the evolutionary and mutational profiles of SARS-CoV-2 variants using serial oropharyngeal swab samples from 5 individuals with persistent infections. Our results revealed that the intra-host evolutionary patterns of different variants varied significantly, and the evolutionary rate in 3 immunocompromised hosts was 20 times higher than in 2 other patients. These variations likely stem from differences in immune suppression status, the strength of preexisting immune responses, and the extent of error-generating mutations. There were 15 intra-host single-nucleotide variants (iSNVs) in the spike gene among at least two variants, suggesting convergent evolution. Although most new iSNVs do not reach fixation, some of them belong to lineage-defined mutations in variants of concern (VOCs) and recent variants of interest (VOIs). The observations indicate that persistent infections serve as sources for novel, potentially harmful variants, whereas the viral evolutionary dynamics are impacted by virological, immunological, and genetic factors. Thus, there is an urgent need for individualized monitoring and management of immunocompromised hosts to prevent outbreaks caused by the viral seeds generated from them and to study viral factors associated with post-acute COVID-19 sequelae.

## 1. Introduction

Novel SARS-CoV-2 variants are always emerging, and they may have increased transmissibility, pathogenicity, and immune escape ability [1]. Immunocompromised hosts generally experience persistent infection of SARS-CoV-2 and may be the hotbed and reservoir of SARS-CoV-2 variants [2,3,4]. Persistent infection gives SARS-CoV-2 prolonged evolutionary time, allowing it to generate immune escape mutations to enhance transmissibility [5,6], and resistance-associated variants could be selected within as little as 10 days of drug exposure [7]. Moreover, the type and extent of immune suppression can impact the viral clearance and intra-host evolutionary diversity of SARS-CoV-2 [8]. In addition, some antiviral drugs, such as RdRp inhibitor molnupiravir, can stop RNA synthesis by introducing G-to-A and C-to-U mutations [9], inferring they may also shape evolutionary patterns, especially for persistent infections, mostly in immunocompromised hosts. Importantly, long COVID occurred more possibly among individuals with persistent infection [10]. However, the potential and mechanism for generating novel variants and their relationship with the post-acute COVID-19 sequelae [11] in persistent SARS-CoV-2 infections remain obscure. Urgently clarifying this link is essential for anticipating the next SARS-CoV-2 outbreak and pinpointing who will develop long COVID-19.

China has unique characteristics in the spread of SARS-CoV-2, especially during the 2022–2023 Omicron wave. This wave, dominated by BA.5.2 and BF.7, occurred against an immunological landscape almost exclusively shaped by wild-type-based vaccines [12]. Most vaccines administered in China were produced using the inactivated wild-type strain as the immunogen [13]. The vaccination schedule is regular in China. By 9 February 2023, fully vaccinated individuals accounted for 89.54% of the total population, and those receiving boosters accounted for 57.99% [14], providing an ideal situation for investigating the viral evolution under a uniform infected and vaccinated immunological background.

In this study, we deep-sequenced serial oropharyngeal swabs from five persistently SARS-CoV-2-infected individuals to quantify the viral intra-host evolutionary rates and track single-nucleotide variant (iSNV) dynamics. These data revealed SARS-CoV-2 within-host evolutionary insights that could guide strategies to avert further outbreaks and mitigate long COVID-19 risk.

## 2. Materials and Methods

### 2.1. Source of Cases

Between May 2023 and October 2023, all inpatients at the First Hospital of China Medical University in Shenyang, a capital city in Northeast China, were recruited if they had a positive SARS-CoV-2 PCR test and then were screened by the inclusion criteria as follows: (1) SARS-CoV-2 PCR test results were positive for at least 30 days; (2) CD4^+^ T cell counts were below the reference lower limit of 410 cells/uL at initiation. For each patient, the following information was collected: antiviral treatment status, vaccination status, prior medical management (including use of immunosuppressive drugs and time since transplant), as well as demographic information (age and sex). Oropharyngeal swabs from patients with a positive SARS-CoV-2 PCR test were collected and stored at 4 °C for RNA isolation and next-generation sequencing. The usage of specimens, as well as the collection of clinical and demographic data, was approved by the Ethical Committee of the First Hospital of China Medical University (Approval No. [2023]331, date: 26 June 2023).

### 2.2. Whole-Genome Sequencing and Data Processing

Viral RNA was extracted from 200 uL of oropharyngeal swabs using a Viral Nucleic Acid Extraction Kit (Magnetic Bead Method) by the automated SSNP-9600A system (BioPerfectus, Taizhou, Jiangsu, China). cDNA synthesis and library preparation were performed using the IDseq^TM^ Complete Plus Multiplex PCR SARS-CoV-2 Genome Kit (Vision Medicals, Guangzhou, Guangdong, China). Then, the amplicon libraries were sequenced on the NextSeq550 instrument (Illunima, San Diego, CA, USA). The raw sequencing data was converted to FASTQ formats, followed by adapter and quality trimming using the BBDuk package in the Geneious Prime software (version 2021.2.2). The clear reads were mapped to the SARS-CoV-2 Wuhan-Hu-1 reference genome (Genbank: NC_045512.2) using the Bowtie2 package in Geneious Prime, and then the consensus sequences were generated based on the >60% base frequency at the simple site. The consensus sequences were used to identify viral lineage according to Pangolin (version 4.1.3). The iSNVs across the spike gene in each sample were defined with a minimum frequency of 0.03 (3%) and a minimum depth of 10 [15].

### 2.3. Phylogenetic Reconstruction

All the associated high-quality genome sequences detected in China, including the variants in this study and their parent lineages, variants of concern (VOCs), and some recent variants of interest (VOIs), were downloaded from GISAID (Table S1). First, the sequences were aligned with MAFFT (version 7.53), and the genomic regions (NC_045512.2: 266-29,674 nt) were retained for phylogenetic reconstruction, which was performed under the HKY + I nucleotide substitution model with 1000 replicates in IQ-Tree (version 1.6.12). The maximum-likelihood tree was visualized using Figtree (version 1.4.4).

### 2.4. Intra-Host Evolution and Diversity Analysis

The intra-host evolutionary rates of the variants for each patient, the population evolutionary rates of the parents’ variants, known VOCs, and related VOIs were calculated under the HKY + I nucleotide substitution model, the relaxed log-normal molecular clock model, and the constant tree prior model in BEAST (version 1.10.4). The Monte Carlo Markov Chain was run until the effective sample size reached 200 after 10% burn-in. The commonalities and differences in the iSNVs were observed and compared based on synonymous and nonsynonymous types across Spike. The temporal mutational dynamics of iSNVs were traced at serial sampling points for each patient and visualized using gradient color.

### 2.5. Statistical Analysis

The accumulation of iSNVs over time was quantified by simple linear regression, and the relationship between iSNV count and intra-host evolutionary rate was assessed with Spearman’s correlation in GraphPad (version 9.51).

## 3. Results

### 3.1. Description of Cases

**Figure 1:**
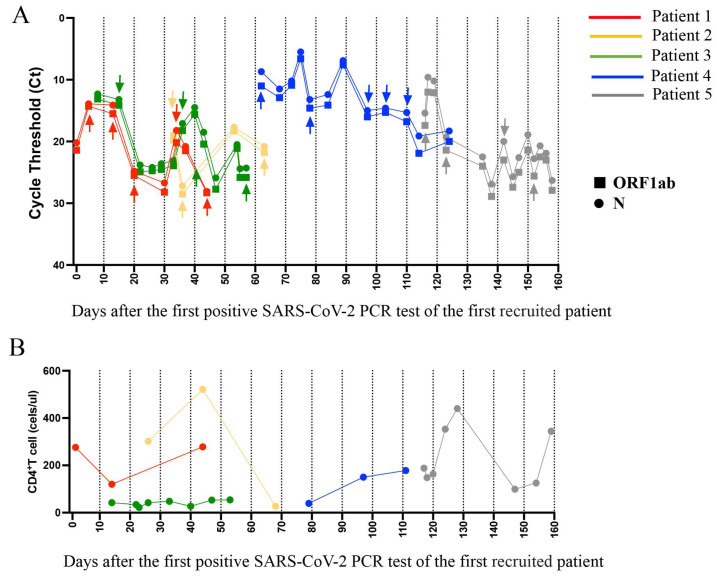
Timeline of virus level and CD4^+^ T cell counts for the five patients. (**A**) The timeline shows the SARS-CoV-2-positive nucleic acid amplification test for five patients. The cycle threshold (Ct) values of ORF1ab and N genes for oropharyngeal swab samples. The arrows represent the sampling time of available genome sequences for analysis. (**B**) Timeline of five patients’ CD4^+^ T cell counts, which can partly reflect the status of immune suppression.

Five individuals with the high-quality SARS-CoV-2 genomes, obtained from serial oropharyngeal swab sequencing, were selected for further evolutionary analysis. The patients consisted of three men and two women, with an average age of 55 (range 37–82). Their medical conditions included kidney transplantation (patients 1 [P1] and 2 [P2]), acute lymphocytic leukemia and stem cell transplantation (P3), and severe infection (P4 and P5) (Table S2). Although the CD4^+^T cell counts for all five patients were below the limit of detection (Figure 1B), the immune conditions of patients P1, P2 and P3 could be classified as immunodeficiency; these conditions may contribute to the persistence and evolution of the virus. In contrast, the impaired immune function of patients P4 and P5 may be primarily due to the severe infection induced by SARS-CoV-2. A minimum of three oropharyngeal swabs were successfully sequenced for each patient, with the average sampling intervals of 41 days (range 32–49). The SARS-CoV-2 PCR CT values for ORF1ab and N genes ranged from 5.5 to 28.2 (Figure 1A). All five patients had received treatment with one or more antiviral drugs, including azvudine, molnupiravir, and Paxlovid (nirmatrelvir–ritonavir) (Figure S1).

### 3.2. Rapid Intra-Host Evolution of SARS-CoV-2 Variants

**Figure 2:**
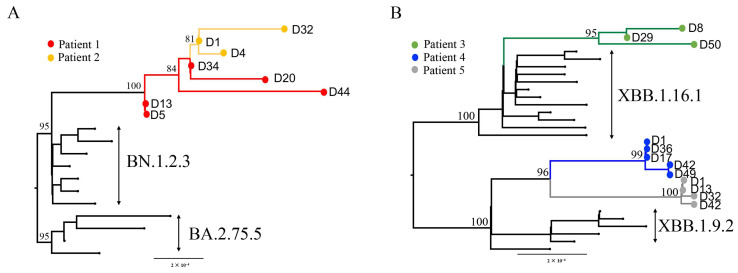
The maximum-likelihood phylogenetic trees of serial samples from each patient reconstructed using the near full-length genome of SARS-CoV-2. Consensus sequences from the five individuals were generated with a base frequency threshold of 60%. These sequences were used to reconstruct the phylogenetic tree with parental reference genomes downloaded from GISAID (Table S1) under HKY + I nucleotide substitution model in IQ-Tree. (**A**) The phylogenetic tree of P1 and P2; (**B**) The phylogenetic tree of P3, P4, and P5.

The SARS-CoV-2 lineages identified in the five patients were FR.1.1 (P1 and P2), FU.1 (P3), EG.5.2.2 (P4), and EG.5.1.1 (P5). Phylogenetic trees, reconstructed from the consensus sequences and their parent reference sequences, revealed intra-host evolution occurred in all five patients (Figure 2). Notably, in P2, three nucleotides in the SARS-CoV-2 genome mutated within just four days. Next, we estimated the intra-host evolutionary rates of these variants in the five patients and compared them to the average evolutionary rate of VOCs and VOIs observed in China (Table 1). The evolutionary rates of the FR.1.1 variant in P1 and P2 were 1.05 × 10^−2^ (95% CI: 7.08 × 10^−3^–1.44 × 10^−2^) and 7.81 × 10^−3^ (95% CI: 3.31 × 10^−3^–1.26 × 10^−2^) nucleotide substitutions per site per year, respectively. In other words, 5.92 (95% CI: 4.00–8.14) and 4.42 (95% CI: 1.87–7.12) nucleotide substitutions per week in P1 and P2, respectively. These rates were nearly 10 times higher than their parental variant BA.2.75, which had a rate of 0.49 [95% CI: 0.33–0.66] nucleotide substitutions per week. The evolutionary rates of FR.1.1 in the above two patients were 8.7 times higher than the B.1.517 variant in an immunocompromised individual who tested positive for SARS-CoV-2 for 471 days, following an allogeneic haploidentical stem cell transplantation in 2019 [16]. Meanwhile, a study of the SARS-CoV-2 intra-host evolution in 94 patients reported that two cases accumulated ≥4.5 nucleotide substitutions over one week [17], similar to the intra-host evolutionary rates of P1 and P2 in this study. For P3, the evolutionary rate of the FU.1 variant was 4.60 × 10^−3^ (95% CI: 2.41 × 10^−3^–6.79 × 10^−3^) nucleotide substitutions per site per year, or 2.60 (95% CI: 1.36–3.84) nucleotide substitutions per week, which was higher than that of its parental variant, CN-XBB.1.16. The other two variants, EG.5.2.2 (P4) and EG.5.1.1 (P5), evolved similarly to their parental variants, XBB.1.19.2 and EG.5, respectively. Notably, the recently emerged variant BA.2.86 evolved more rapidly than VOCs and some VOIs. However, the intra-host evolutionary rates of FR.1.1 (P1 and P2) and FU.1 (P3) were still higher than the upper 95% CI of the estimated rate of BA.2.86.

### 3.3. The Increased Virus Genetic Diversity and Variable iSNV Characteristics

Observing the significant differences in intra-host evolutionary rates among the 5 patients, we hypothesized that virus genetic diversity and iSNV dynamics would also vary. We defined iSNV with its frequency above 0.03 in 1 sample according to the next-generation sequencing data and analyzed the changes in the number and characteristics of iSNVs over time throughout the *spike* gene, which can be heavily influenced by immune selection.

**Figure 3:**
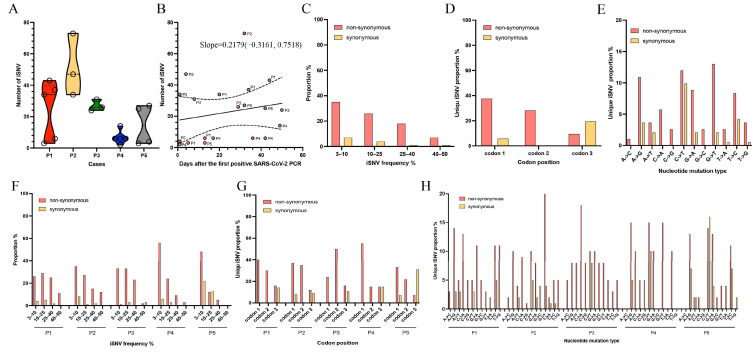
The intra-host evolutionary diversity and variable iSNV characteristics. (**A**) The number of >3% iSNV was listed for each patient across all sampling times during persistent infection. (**B**) The relationship between the genetic diversity and sampling time was estimated for all samples from the five patients using linear regression. (**C**–**E**) The proportions of iSNV at different frequencies, codon positions and mutational types for all samples from the five patients. (**F**–**H**) The proportions of iSNV at different frequencies, codon positions and mutational types for all samples from each patient.

Overall, for the 5 individuals, the number of iSNVs over time increased under linear regression (slope = 0.2179), which varied per sample per case (mean: 22.55, range: 2–73) (Figure 3A,B). P1, P2 and P3 had more iSNVs than P4 and P5; this finding was consistent with the difference in intra-host evolutionary rate, as estimated by the consensus genomes (Figure 3A; Table 1). Additionally, the correlation analysis indicated that the number of iSNVs, especially those at high frequency, is positively correlated to the evolutionary rate (*p* = 0.0114) (Figure S2A,B). We analyzed nonsynonymous and synonymous mutations across frequency ranges of 3–10%, 10–25%, 25–40%, and 40–50% and found that the nonsynonymous iSNVs consistently predominated, but their proportions declined as iSNV frequencies increased (Figure 3C). In P1, P2, and P3, the patterns of nonsynonymous and synonymous mutations at various iSNV frequencies were similar; however, in P4 and P5, the iSNVs with frequencies of 3–10 were predominant (Figure 3F).

We further investigated the impacts of >3% iSNV on viral evolution, focusing on different codon positions and nucleotide types. Distinguishing the >3% iSNVs by codon position, we found that the nonsynonymous iSNVs predominated at the first and second codon positions, accounting for 65.63% (Figure 3D). We also observed that most synonymous iSNVs were located at the third codon position, and these were more than nonsynonymous iSNVs (19.27% vs. 9.38%, respectively) (Figure 3D). However, the codon patterns varied between individuals. In P1, P2, and P3, the proportion of synonymous iSNVs at the third codon was lower than that of nonsynonymous iSNVs (Figure 3G). When the iSNVs were stratified by nucleotide types, >3% of iSNVs contained relatively more C-to-T substitutions (22%), with similar proportions of nonsynonymous and synonymous mutations (Figure 3E). Other nucleotide types, such as A-to-G, G-to-A, G-to-T and T-to-C, which accounted for more than 10% of iSNVs, contained more nonsynonymous mutations (Figure 3E). Similarly, the nucleotide substitution types varied by patient. Specifically, A-to-G (17%) and T-to-C (16%) predominated in P1, G-to-T (27%) and C-to-T (15%) in P2, C-to-A (18%) and C-to-T (18%) in P3, C-to-T (25%) and A-to-G (20%) in P4, and C-to-T (30%) and A-to-G (20%) in P5 (Figure 3H). Notably, the C-to-T substitution, which is associated with the natural antiviral host response induced by APOBEC family enzymes, showed a higher proportion of synonymous substitutions in P4 and P5, and these patients exhibited slower viral evolution (Figure 3H and Table 1). Interestingly, the proportions of C-to-T were negatively correlated to intra-host evolutionary rates (*p* = 0.0333) (Figure S2C).

### 3.4. The Temporal Dynamics of iSNV Mutational Types Induced by Convergent Evolution and Negative Selection

**Figure 4:**
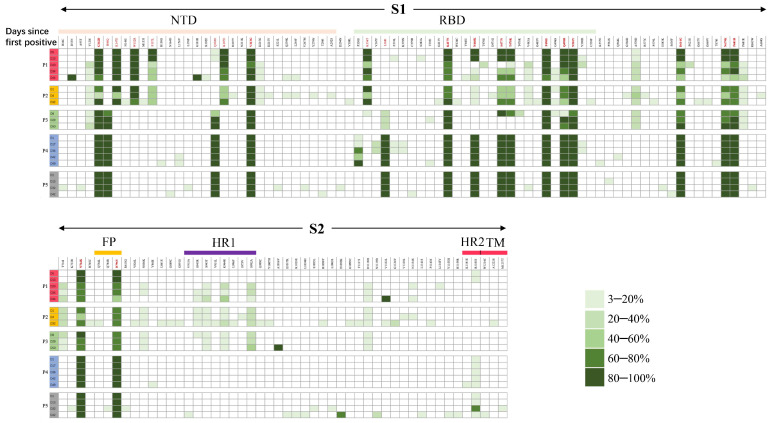
The temporal mutational dynamics of iSNV in the spike gene. The frequency changes and the types of amino acid substitution of iSNVs throughout the SARS-CoV-2 *spike* gene were compared with the Wuhan-Hu-1 reference genome (Genbank: NC_045512.2). Amino acid substitutions highlighted in red represent the Pango lineage-defined mutations, while bold substitutions indicate mutations detected in more than two different lineages.

We compared the changes in amino acid mutations in the spike protein over time to better understand the selective advantage of SARS-CoV-2 in persistent infection (Figure 4). First, we observed seven mutations (D53N, G181R, P330S, A1016V, E1092K, V1122L, R1185S) that appeared in P1, P3, P4, and P5. These mutations rapidly increased to frequencies above 60%. D53N and G181R are located in the NTD region, P330S is in the RBD region, and R1185S is in the HR2 region. In contrast, the frequencies of ten Pango lineage-defined mutations (F157L, K187E, S477N, T478K, Q498R, D614G, N679K, P681H, N764K and D796Y) declined over time, with several eventually dropping to zero. Second, 15 intra-host iSNVs, including V120I, F374L, T385I, V483A, G496S, Y508H, A570D, P681R, T716I, N856K, S943R, S943T, K964R, S982A, D1118H and R1185S, occurred in at least two lineages. Among these iSNVs, one mutation (V120I) is in the NTD region, four (F374L, T385I, V483A, G596S) in the RBD region, four (S943R, S943T, K964R, S982A) in the HR1 region, and one (R1185S) in the HR2 region. Finally, most iSNVs fluctuated at low frequencies and did not reach fixation, rising temporarily before decreasing again. However, 16 iSNVs, including V120I, L176V, D215G, P330S, L368I, V483A, G496S, A570D, P681R, T716I, N856K, S943R, S943T, K964R, S982A, D1118H, and R1185S, persisted after their initial occurrence. Thirteen of them were found in more than 2 lineages. These findings demonstrate that both convergent evolution and negative selection contribute to shaping the viral genetic diversity.

### 3.5. Mutations Related to Antiviral Drugs

All five patients received antiviral drug treatments; however, SARS-CoV-2 remained detectable for at least 30 days. We further analyzed the occurrence of drug resistance and drug-related mutations. No known resistance mutations associated with Paxlovid [18,19], which was used in P3, P4, and P5, were observed during prolonged SARS-CoV-2 infection. Additionally, the proportions of G-to-A mutations, which are linked to RNA synthesis inhibition by RdRp-targeting drugs like molnupiravir, used for all five patients, were 10.78% in P1, 10.20% in P2, 9.62% in P3, 10% in P4, and 16.50% in P5.

## 4. Discussion

In this study, through comprehensive genomic analysis, we have revealed that the intra-host evolutionary patterns of SARS-CoV-2 variants differ in persistent infections across individuals with similar preexisting immune conditions but distinct immunosuppressive statuses. The key observations include the variations in intra-host evolutionary rates, with differences reaching up to 20 times. Additionally, the types of mutations in iSNVs varied, particularly those related to error-generating mutations. These findings emphasize that monitoring the generation of novel variants should not focus solely on persistent infections, and greater attention should also be given to the type of immunodeficiency, the variants’ response to individual immune systems, and the extent of coding errors induced by antiviral host mechanisms.

First, the rate of SARS-CoV-2 evolution varied among the patients. Accelerated evolution of SARS-CoV-2 was observed in three patients with a history of organ or stem cell transplantation, as well as long-term immunosuppressant use. In these patients, the intra-host evolutionary rate was 4–10 times faster than that of the parent variants. In contrast, the intra-host evolution of SARS-CoV-2 in two patients with severe infection was similar to the population-level evolution of the parent variants. This finding is consistent with previous studies suggesting that the severity of immunocompromised conditions can influence viral evolution [8].

Second, preexisting immune responses appear to influence SARS-CoV-2 evolution. In China, inactivated COVID-19 vaccines were widely designed for ancestral SARS-CoV-2, and some protein subunit vaccines were developed for some variants [13,20]. On December 7, 2022, China adjusted its public health control measures for COVID-19, leading to an epidemic in early 2023, primarily driven by Omicron variants BA.5.2 and BF.7 [21]. The preexisting immune responses from vaccination and breakthrough infections were believed to impose varying pressures on the variants, thereby driving viral evolution. Dai et al. compared the neutralizing abilities of the serum samples from individuals vaccinated with the wild-type inactivated vaccine after the BA.5.2 and BF.7 outbreaks. They found significantly higher neutralizing titers against the ancestral strain, Delta, BA.5.2, BF.7, and BA.2.75, compared to the XBB, BQ.1, and BQ.1.1 variants [12]. Similarly, our team analyzed the binding abilities of serum IgG from HIV-positive individuals 4 to 6 months after the BA.5.2 and BF.7 outbreaks, and we found a substantial reduction in binding abilities against EG.5.1 and XBB.1.9, followed by XBB.1.16 and BA.2 (unpublished data). This suggests that the viruses in P1 and P2, infected by descendant variants of BA.2.75, might have generated escape mutations under strong immune pressure from breakthrough infections of BA.5.2 and BF.7, resulting in more mutations. In contrast, the viruses in P3, P4 and P5, infected by descendant variants of XBB.1.16 and EG.5/XBB.1.19, may have been under moderate or weak immune pressure, leading to fewer mutations. The varying patterns of iSNVs between patients further support the idea that the variants infecting these five patients were under different immune selections. Thus, the strong or moderate immune pressures induced by preexisting immune responses in P1, P2, and P3 may have accelerated the intra-host evolution of SARS-CoV-2 variants.

Third, host factors may influence SARS-CoV-2 evolution. Host factors can attack RNA virus genomes by inducing error-generating mutations or altering viral fitness through changes in codon usage preference [22,23,24]. For example, the apolipoprotein B mRNA editing enzyme catalytic polypeptide-like family enzyme (APOBEC) can induce C-to-U mutations, while the adenosine deaminase acting on RNA family enzyme (ADAR) can induce A-to-G mutations. Both of these mutations have the potential to inhibit RNA synthesis by producing stop codons [25,26]. Additionally, some antiviral drugs targeting SARS-CoV-2 RNA-dependent RNA polymerase (RdRp) can block RNA synthesis by inducing transition errors, such as AT to GC, as seen with molnupiravir [27]. A systematic analysis of the GISAID database found a high proportion of G-to-A and C-to-U mutations after 2022, when molnupiravir was widely used in some countries or specific age groups [28]. In our study, C-to-T mutation predominated, consistent with population-level findings [22]. However, the types of nucleotide mutations differed between patients. The proportions of C-to-T mutations were higher with P4 and P5 compared to P1, P2 and P3. Moreover, all five patients received molnupiravir treatment, and the proportions of G-to-A were similar across all individuals, except for P5. This suggests that the influence of antiviral host factors was stronger for P4 and P5 than for P1, P2 and P3. These error-generating mutations may reduce the viral quasispecies through multiple rounds of replication, potentially explaining the slower evolution of SARS-CoV-2 variants in P4 and P5.

Lastly, the *spike* gene plays a critical role in SARS-CoV-2 evolution. The *spike* gene is the core target for viral attachment, entry and immune escape [29]. We identified several iSNVs in the functional domains of the *spike* gene, some with high frequencies (D53N and G181R in the NTD; P330S in the RBD; R1185S in the HR2) and others that persisted at low frequencies across different lineages (V120I in the NTD; G496S in the RBD; S943T/R, K964R, and S982A in the HR1; R1185S in the HR2). These iSNVs are thought to enhance viral fitness or undergo immune selection. For example, G496S increases ACE2 binding activity and reduces the neutralizing activity of mAb [30,31], while S982A enhances viral entry and hinders the induction of cross-neutralizing antibodies [32]. The generation of iSNVs across lineages under various immune conditions further highlights the role of convergent evolution in persistent infections and the advantage of these iSNVs in viral fitness. Our observation that the most iSNVs exhibited random fluctuation suggests the significant role of neutral evolution. While these iSNVs often disappeared due to negative selection, some have been observed in VOCs, such as T716I, S982A, and D1118H in VOC Alpha; D215G in VOC Beta; and P681R in VOC Delta. Notably, some iSNVs, including R158G, K356T, A570V, P621S, P681R, and P1143L, have also been detected in recent VOIs such as BA.2.86 and JN.1, which emerged after our study was completed. Overall, the temporal mutational dynamics of *spike* gene iSNVs in the five patients with persistent infection suggest that convergent evolution and negative selection act together in generating novel variants. These variants may provide advantages in viral transmission and immune escape.

This study has some limitations. First, further testing of humoral and cellular immunity responses, such as measuring SARS-CoV-2-specific antibody titers and T cell proliferation, is necessary to verify the phenotype associated with the developed mutation. Second, since the patients were in different statuses of immune suppression, additional studies are required to explore the relationship between immune suppression and evolutionary diversity in a larger cohort.

## 5. Conclusions

Our findings suggest that while immunocompromised hosts may serve as sources of novel SARS-CoV-2 variants, they still require tailored monitoring and management based on their virological and immunological conditions. Furthermore, it is crucial to focus on the recovery and treatment of persistent infections to prevent long COVID.

## Figures and Tables

**Table 1:** The intra-host evolutionary rates of SARS-CoV-2 variants and populational evolutionary rates of their parental variants, VOCs and recently emerged VOIs.

Variant	Mutations per Week (s/week)			Mutations per Site per Year (s/s/y)		
	Median	Lower 95% CI	Upper 95% CI	Median	Lower 95% CI	Upper 95% CI
P1	5.92	4.00	8.14	1.05 × 10^−2^	7.08 × 10^−3^	1.44 × 10^−2^
P2	4.42	1.87	7.12	7.81 × 10^−3^	3.31 × 10^−3^	1.26 × 10^−2^
P3	2.60	1.36	3.84	4.60 × 10^−3^	2.41 × 10^−3^	6.79 × 10^−3^
P4	0.31	0.04	0.78	5.55 × 10^−4^	7.12 × 10^−5^	1.38 × 10^−3^
P5	0.31	0.21	0.85	5.44 × 10^−4^	3.76 × 10^−4^	1.51 × 10^−3^
CN-Alpha	0.58	0.08	1.05	1.02 × 10^−3^	1.44 × 10^−4^	1.85 × 10^−3^
CN-Beta	0.33	0.00	1.05	5.81 × 10^−4^	2.23 × 10^−6^	1.39 × 10^−3^
CN-Gamma	0.51	0.00	2.22	9.00 × 10^−4^	3.12 × 10^−6^	3.92 × 10^−3^
CN-Delta	0.19	0.11	0.29	3.37 × 10^−4^	1.96 × 10^−4^	5.14 × 10^−4^
CN-Omicron	0.38	0.31	0.46	6.67 × 10^−4^	5.44 × 10^−4^	8.17 × 10^−3^
CN-BA.2.86	0.81	0.11	1.54	1.43 × 10^−3^	1.89 × 10^−4^	2.72 × 10^−3^
CN-JN.1	0.40	0.06	0.68	7.08 × 10^−4^	1.27 × 10^−4^	1.21 × 10^−3^
CN-BA.2.75	0.49	0.33	0.66	8.61 × 10^−4^	5.80 × 10^−4^	1.17 × 10^−3^
CN-XBB.1.16	0.60	0.01	1.48	1.06 × 10^−3^	8.72 × 10^−6^	2.62 × 10^−3^
CN-XBB.1.19.2	0.41	0.07	1.00	7.26 × 10^−4^	1.31 × 10^−4^	1.76 × 10^−3^
CN-EG.5	0.30	0.23	0.38	5.26 × 10^−4^	4.05 × 10^−4^	6.68 × 10^−4^

## Data Availability

The raw data generated through next-generation sequencing in this study is available in the National Microbiology Data Center (https://nmdc.cn/resource/genomics/sra) as fastq. files under the bioproject name NMDC10018864 (https://nmdc.cn/submit/project/overview/SUB1714275784345) (accessed on 10 April 2025). The sequences are accessible with the following access numbers: NMDC40055081, NMDC400550843-NMDC4005086 for patient 1; NMDC40055077-NMDC40055079 for patient 2; NMDC40055090-NMDC40055092 for patient 3; NMDC40055093-NMDC40055097 for patient 4; and NMDC40055098-NMDC40055099, NMDC40055101-NMDC40055102 for patient 5.

## Supplementary Materials

The following supporting information can be downloaded at: https://www.mdpi.com/article/10.3390/microorganisms13112613/s1, Figure S1: Timeline of SARS-CoV-2 antiviral drug usages of five patients; Figure S2: The relationship between iSNV and intra-host evolutionary rate analyzed using spearman’s correlation; Table S1: The reference sequences from GISAID used in this study; Table S2: Summary of the five patients’ demographic data and medical history.

## References

[1] Carabelli A.M., Peacock T.P., Thorne L.G., Harvey W.T., Hughes J., Peacock, Barclay W.S., de Silva T.I., Towers G.J., COVID-19 Genomics UK Consortium (2023). SARS-CoV-2 variant biology: Immune escape, transmission and fitness. Nat. Rev. Microbiol..

[2] Harari S., Tahor M., Rutsinsky N., Meijer S., Miller D., Henig O., Halutz O., Levytskyi K., Ben-Ami R., Adler A. (2022). Drivers of adaptive evolution during chronic SARS-CoV-2 infections. Nat. Med..

[3] Burki T. (2022). The origin of SARS-CoV-2 variants of concern. Lancet Infect. Dis..

[4] Weigang S., Fuchs J., Zimmer G., Schnepf D., Kern L., Beer J., Luxenburger H., Ankerhold J., Falcone V., Kemming J. (2021). Within-host evolution of SARS-CoV-2 in an immunosuppressed COVID-19 patient as a source of immune escape variants. Nat. Commun..

[5] Choi B., Choudhary M.C., Regan J., Sparks J.A., Padera R.F., Qiu X., Solomon I.H., Kuo H.-H., Boucau J., Bowman K. (2020). Persistence and Evolution of SARS-CoV-2 in an Immunocompromised Host. N. Engl. J. Med..

[6] Machkovech H.M., Hahn A.M., Garonzik Wang J., Grubaugh N.D., Halfmann P.J., Johnson M.C., Lemieux J.E., O’Connor D.H., Piantadosi A., Wei W. (2024). Persistent SARS-CoV-2 infection: Significance and implications. Lancet. Infect. Dis..

[7] Fountain-Jones N.M., Vanhaeften R., Williamson J., Maskell J., Chua I.-L.J., Charleston M., Cooley L. (2024). Effect of molnupiravir on SARS-CoV-2 evolution in immunocompromised patients: A retrospective observational study. Lancet Microbe.

[8] Li Y., Choudhary M.C., Regan J., Boucau J., Nathan A., Speidel T., Liew M.Y., Edelstein G.E., Kawano Y., Uddin R. (2024). SARS-CoV-2 viral clearance and evolution varies by type and severity of immunodeficiency. Sci. Transl. Med..

[9] Li G., Hilgenfeld R., Whitley R., De Clercq E. (2023). Therapeutic strategies for COVID-19: Progress and lessons learned. Nat. Rev. Drug Discov..

[10] Ghafari M., Hall M., Golubchik T., Ayoubkhani D., House T., MacIntyre-Cockett G., Fryer H.R., Thomson L., Nurtay A., Kemp S.A. (2024). Prevalence of persistent SARS-CoV-2 in a large community surveillance study. Nature.

[11] Davis H.E., McCorkell L., Vogel J.M., Topol E.J. (2023). Long COVID: Major findings, mechanisms and recommendations. Nat. Rev. Microbiol..

[12] Dai L., Duan H., Liu X., Zhou H., Duan M., An Y., Yuan L., Zhao X., Xu K., Wu Q. (2023). Omicron neutralisation: RBD-dimer booster versus BF.7 and BA.5.2 breakthrough infection. Lancet.

[13] Xu K., Fan C., Han Y., Dai L., Gao G.F. (2022). Immunogenicity, efficacy and safety of COVID-19 vaccines: An update of data published by 31 December 2021. Int. Immunol..

[14] Mathieu E., Ritchie H., Rodés-Guirao L., Appel C., Giattino C., Hasell J., Macdonald B., Dattani S., Beltekian D., Ortiz-Ospina E. (2020). Coronavirus Pandemic (COVID-19). Our World in Data. https://ourworldindata.org/covid-vaccinations.

[15] Álvarez H., Ruiz-Mateos E., Juiz-González P.M., Vitallé J., Viéitez I., Vázquez-Friol M.D.C., Torres-Beceiro I., Pérez-Gómez A., Gallego-García P., Estévez-Gómez N. (2022). SARS-CoV-2 Evolution and Spike-Specific CD4+ T-Cell Response in Persistent COVID-19 with Severe HIV Immune Suppression. Microorganisms.

[16] Chaguza C., Hahn A.M., Petrone M.E., Zhou S., Ferguson D., Breban M.I., Pham K., Peña-Hernández M.A., Castaldi C., Hill V. (2023). Accelerated SARS-CoV-2 intrahost evolution leading to distinct genotypes during chronic infection. Cell Rep. Med..

[17] Landis J.T., Moorad R., Pluta L.J., Caro-Vegas C., McNamara R.P., Eason A.B., Bailey A., Villamor F.C.S., Juarez A., Wong J.P. (2023). Intra-Host Evolution Provides for the Continuous Emergence of SARS-CoV-2 Variants. mBio.

[18] Iketani S., Mohri H., Culbertson B., Hong S.J., Duan Y., Luck M.I., Annavajhala M.K., Guo Y., Sheng Z., Uhlemann A.-C. (2023). Multiple pathways for SARS-CoV-2 resistance to nirmatrelvir. Nature.

[19] Bouzidi H.S., Driouich J.-S., Klitting R., Bernadin O., Piorkowski G., Amaral R., Fraisse L., Mowbray C.E., Scandale I., Escudié F. (2024). Generation and evaluation of protease inhibitor-resistant SARS-CoV-2 strains. Antivir. Res..

[20] Dai L., Gao L., Tao L., Hadinegoro S.R., Erkin M., Ying Z., He P., Girsang R.T., Vergara H., Akram J. (2022). Efficacy and Safety of the RBD-Dimer-Based Covid-19 Vaccine ZF2001 in Adults. N. Engl. J. Med..

[21] Sun Y., Wang M., Lin W., Dong W., Xu J. (2023). Evolutionary analysis of Omicron variant BF.7 and BA.5.2 pandemic in China. J. Biosaf. Biosecurity.

[22] Wu X., Shan K.-J., Zan F., Tang X., Qian Z., Lu J. (2023). Optimization and Deoptimization of Codons in SARS-CoV-2 and Related Implications for Vaccine Development. Adv. Sci..

[23] Duffy S., Shackelton L.A., Holmes E.C. (2008). Rates of evolutionary change in viruses: Patterns and determinants. Nat. Rev. Genet..

[24] Shen X., Song S., Li C., Zhang J. (2022). Synonymous mutations in representative yeast genes are mostly strongly non-neutral. Nature.

[25] Shan K.-J., Wei C., Wang Y., Huan Q., Qian W. (2021). Host-specific asymmetric accumulation of mutation types reveals that the origin of SARS-CoV-2 is consistent with a natural process. Innovation.

[26] Deng S., Xing K., He X. (2022). Mutation signatures inform the natural host of SARS-CoV-2. Natl. Sci. Rev..

[27] Kabinger F., Stiller C., Schmitzová J., Dienemann C., Kokic G., Hillen H.S., Höbartner C., Cramer P. (2021). Mechanism of molnupiravir-induced SARS-CoV-2 mutagenesis. Nat. Struct. Mol. Biol..

[28] Sanderson T., Hisner R., Donovan-Banfield I., Hartman H., Løchen A., Peacock T.P., Ruis C. (2023). A molnupiravir-associated mutational signature in global SARS-CoV-2 genomes. Nature.

[29] Harvey W.T., Carabelli A.M., Jackson B., Gupta R.K., Thomson E.C., Harrison E.M., Ludden C., Reeve R., Rambaut A., COVID-19 Genomics UK (COG-UK) Consortium (2021). SARS-CoV-2 variants, spike mutations and immune escape. Nat. Rev. Microbiol..

[30] Cao Y., Wang J., Jian F., Xiao T., Song W., Yisimayi A., Huang W., Li Q., Wang P., An R. (2022). Omicron escapes the majority of existing SARS-CoV-2 neutralizing antibodies. Nature.

[31] Khan A., Khan S.A., Zia K., Altowyan M.S., Barakat A., Ul-Haq Z. (2022). Deciphering the Impact of Mutations on the Binding Efficacy of SARS-CoV-2 Omicron and Delta Variants With Human ACE2 Receptor. Front. Chem..

[32] Peng Q., Zhou R., Liu N., Wang H., Xu H., Zhao M., Yang D., Au K.-K., Huang H., Liu L. (2022). Naturally occurring spike mutations influence the infectivity and immunogenicity of SARS-CoV-2. Cell. Mol. Immunol..

