# Supplementary material for: Immune Suppression, Preexisting Immunity, and Mutation Tendency Shaped SARS-CoV-2 Evolution in Persistent Infection
Source: Microorganisms. 2025 Nov 17;13(11):2613. doi: 10.3390/microorganisms13112613 (PMC12654367; doi:10.3390/microorganisms13112613)
Supplement: Supplementary file 1 [file microorganisms-13-02613-s001.zip › microorganisms-3903115-supplementary.pdf]

Supplementary Materials

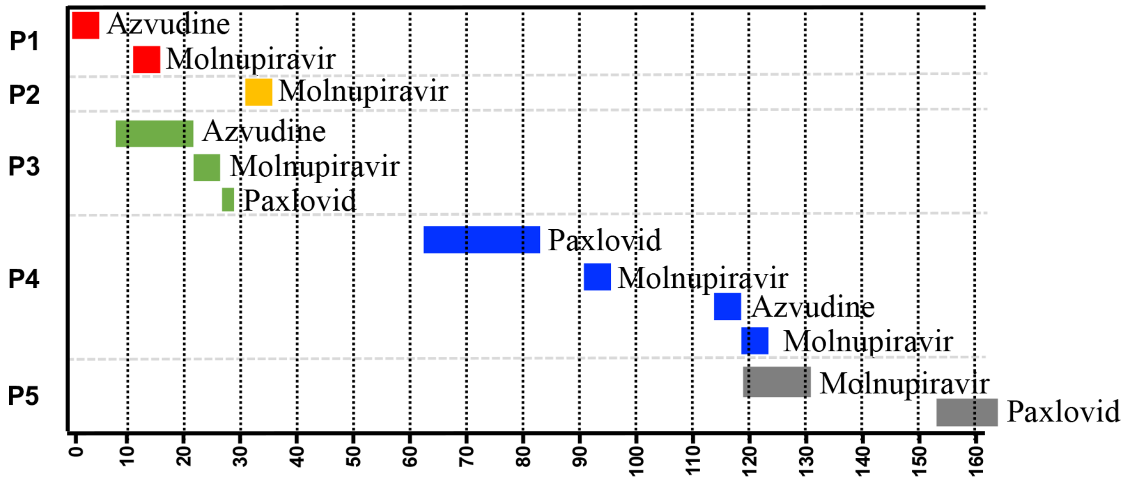

Figure S1. Timeline of SARS-CoV-2 antiviral drug usages of five patients.

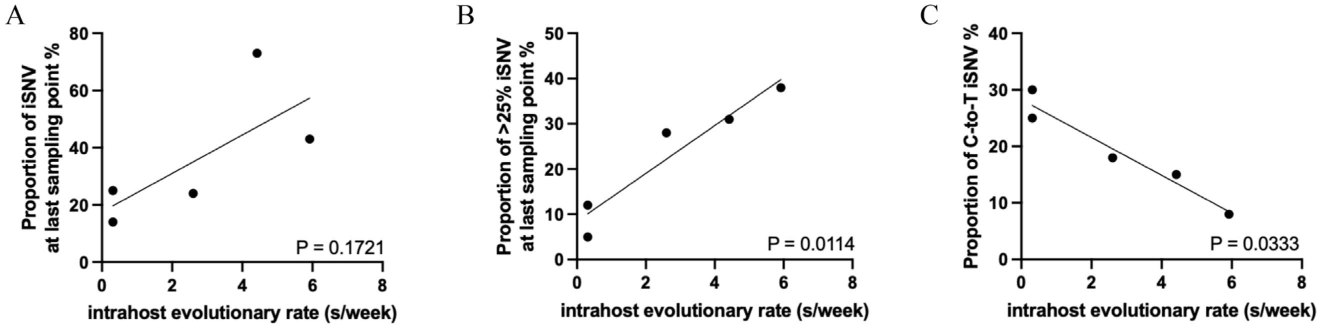

Figure S2. The relationship between iSNV and intra-host evolutionary rate analyzed using spearman's correlation.

Table S1. The reference sequences from GISAID used in this study.

GISAID number	GISAID number	GISAID number	GISAID number	GISAID number
EPI_ISL_17672013	EPI_ISL_16568529	EPI_ISL_2432956	EPI_ISL_15756088	EPI_ISL_3127444
EPI_ISL_17729935	EPI_ISL_16584587	EPI_ISL_2432957	EPI_ISL_15756108	EPI_ISL_3611060
EPI_ISL_17801851	EPI_ISL_16679626	EPI_ISL_2500849	EPI_ISL_15756110	EPI_ISL_7876605
EPI_ISL_17972977	EPI_ISL_16714990	EPI_ISL_8582073	EPI_ISL_15756121	EPI_ISL_8525421
EPI_ISL_17972983	EPI_ISL_17825830	EPI_ISL_8582079	EPI_ISL_15756126	EPI_ISL_8540924
EPI_ISL_18138319	EPI_ISL_16702919	EPI_ISL_8582080	EPI_ISL_15756127	EPI_ISL_9910252
EPI_ISL_18495203	EPI_ISL_16837048	EPI_ISL_13499640	EPI_ISL_15775694	EPI_ISL_9910289
EPI_ISL_18495253	EPI_ISL_16900007	EPI_ISL_18934690	EPI_ISL_15775706	EPI_ISL_18070465
EPI_ISL_18495274	EPI_ISL_17562218	EPI_ISL_18934693	EPI_ISL_16138066	EPI_ISL_18070466
EPI_ISL_18495284	EPI_ISL_1121993	EPI_ISL_2779639	EPI_ISL_16138170	EPI_ISL_18070467
EPI_ISL_18495311	EPI_ISL_1122015	EPI_ISL_2931278	EPI_ISL_16138216	EPI_ISL_18070468

EPI_ISL_18495339	EPI_ISL_2405168	EPI_ISL_8582083	EPI_ISL_16138277	EPI_ISL_18070469
EPI_ISL_17487882	EPI_ISL_2405169	EPI_ISL_8582087	EPI_ISL_16138280	EPI_ISL_18070470
EPI_ISL_17684238	EPI_ISL_2405170	EPI_ISL_10925297	EPI_ISL_16610212	EPI_ISL_18070471
EPI_ISL_17769966	EPI_ISL_2405171	EPI_ISL_10925418	EPI_ISL_17261504	EPI_ISL_18070472
EPI_ISL_17816976	EPI_ISL_2405172	EPI_ISL_10925444	EPI_ISL_17261537	EPI_ISL_18070473
EPI_ISL_17837039	EPI_ISL_2405173	EPI_ISL_12030362	EPI_ISL_17261578	EPI_ISL_18070475
EPI_ISL_18495226	EPI_ISL_2405175	EPI_ISL_12030372	EPI_ISL_1911197	EPI_ISL_18070476
EPI_ISL_16493914	EPI_ISL_2405176	EPI_ISL_13008837	EPI_ISL_1911250	EPI_ISL_18070477
EPI_ISL_16493961	EPI_ISL_2432955	EPI_ISL_13008967	EPI_ISL_2723563	EPI_ISL_18070478
EPI_ISL_11799970	EPI_ISL_16614641	EPI_ISL_16926323	EPI_ISL_18111219	EPI_ISL_18853593
EPI_ISL_11799984	EPI_ISL_16614708	EPI_ISL_17045277	EPI_ISL_18146021	EPI_ISL_18916153
EPI_ISL_11873910	EPI_ISL_16614718	EPI_ISL_17197592	EPI_ISL_18313096	EPI_ISL_18916193
EPI_ISL_11873918	EPI_ISL_16633988	EPI_ISL_17261637	EPI_ISL_18330215	EPI_ISL_18941285
EPI_ISL_11873919	EPI_ISL_16634088	EPI_ISL_17485741	EPI_ISL_18388906	EPI_ISL_18982897
EPI_ISL_11873930	EPI_ISL_16634135	EPI_ISL_17487900	EPI_ISL_18438423	EPI_ISL_18982910
EPI_ISL_11873938	EPI_ISL_16641868	EPI_ISL_17487959	EPI_ISL_18485280	EPI_ISL_18982938
EPI_ISL_12030359	EPI_ISL_16641980	EPI_ISL_17488017	EPI_ISL_18495191	EPI_ISL_18983018
EPI_ISL_12241082	EPI_ISL_16642025	EPI_ISL_17684223	EPI_ISL_18495232	EPI_ISL_18647486
EPI_ISL_13858940	EPI_ISL_16679341	EPI_ISL_17684237	EPI_ISL_18495306	EPI_ISL_18797881
EPI_ISL_13858961	EPI_ISL_16723141	EPI_ISL_17697931	EPI_ISL_18495411	EPI_ISL_18853598
EPI_ISL_16327373	EPI_ISL_16829061	EPI_ISL_17729889	EPI_ISL_18495470	EPI_ISL_18916149
EPI_ISL_16327420	EPI_ISL_16829062	EPI_ISL_17729935	EPI_ISL_18602685	EPI_ISL_18916157
EPI_ISL_16327645	EPI_ISL_16922276	EPI_ISL_17794122	EPI_ISL_18602775	EPI_ISL_18797878
EPI_ISL_16390996	EPI_ISL_16922381	EPI_ISL_17794152	EPI_ISL_18608349	EPI_ISL_18797884
EPI_ISL_16604725	EPI_ISL_16922726	EPI_ISL_17794244	EPI_ISL_18647501	EPI_ISL_18797889
EPI_ISL_16610553	EPI_ISL_16923678	EPI_ISL_17978556	EPI_ISL_18797876	EPI_ISL_18853591
EPI_ISL_16614188	EPI_ISL_16923710	EPI_ISL_18070356	EPI_ISL_18797890	EPI_ISL_18853602
EPI_ISL_16614373	EPI_ISL_16923853	EPI_ISL_18070482	EPI_ISL_18797892	EPI_ISL_18853605
EPI_ISL_16614552	EPI_ISL_16923947	EPI_ISL_18095800	EPI_ISL_18805117	EPI_ISL_18916153
EPI_ISL_18961247	EPI_ISL_17197592	EPI_ISL_17769966	EPI_ISL_18078278	EPI_ISL_18983029
EPI_ISL_18982901	EPI_ISL_17273688	EPI_ISL_17794126	EPI_ISL_18114926	EPI_ISL_18983038
EPI_ISL_18982929	EPI_ISL_17489837	EPI_ISL_17794212	EPI_ISL_18254205	EPI_ISL_18983041
EPI_ISL_18982934	EPI_ISL_17489852	EPI_ISL_17794257	EPI_ISL_18313198	
EPI_ISL_18983014	EPI_ISL_17494023	EPI_ISL_17816976	EPI_ISL_18376459	
EPI_ISL_18983022	EPI_ISL_17494087	EPI_ISL_17837035	EPI_ISL_18401765	
EPI_ISL_16327334	EPI_ISL_17511947	EPI_ISL_17837039	EPI_ISL_18401875	
EPI_ISL_16327621	EPI_ISL_17584693	EPI_ISL_18006125	EPI_ISL_18438359	
EPI_ISL_16614676	EPI_ISL_17637396	EPI_ISL_18037054	EPI_ISL_18495492	
EPI_ISL_16614733	EPI_ISL_17671893	EPI_ISL_18049872	EPI_ISL_18589885	
EPI_ISL_16641967	EPI_ISL_17672009	EPI_ISL_18245925	EPI_ISL_18602732	
EPI_ISL_16642025	EPI_ISL_17684230	EPI_ISL_18495226	EPI_ISL_18602759	
EPI_ISL_16713273	EPI_ISL_17697738	EPI_ISL_17696901	EPI_ISL_18608349	
EPI_ISL_16750666	EPI_ISL_17794121	EPI_ISL_17697639	EPI_ISL_18805117	

EPI_ISL_16750702	EPI_ISL_17988074	EPI_ISL_17801823	EPI_ISL_18805122	
EPI_ISL_16854466	EPI_ISL_17988886	EPI_ISL_17962823	EPI_ISL_18805124	
EPI_ISL_16922506	EPI_ISL_17487882	EPI_ISL_17978549	EPI_ISL_18853600	
EPI_ISL_16922525	EPI_ISL_17684238	EPI_ISL_17988888	EPI_ISL_18941282	
EPI_ISL_17170729	EPI_ISL_17684257	EPI_ISL_18037123	EPI_ISL_18961218	
EPI_ISL_17170841	EPI_ISL_17697961	EPI_ISL_18078251	EPI_ISL_18982899	

Table S2. Summary of the five patients' demographic data and medical history.

**Patient**	**Sex**	**Age**	**Medical history before this SARS-CoV-2 diagnosis**	**First time of positive SARS-CoV-2 PCR test**	**SARS-CoV-2 Lineage**
P1	Male	47	2021-6-23 Kidney transplantation, anti-rejection by Tacrolimus<br>2023-5-16 Diagnosis of Aspergillus infection	2023/5/4	FR.1.1
P2	Female	49	2021-11-24 Kidney transplantation, anti-rejection by Tacrolimus<br>2023-5-29 Diagnosis of Kidney allograft dysfunction	2023/6/5	FR.1.1
P3	Female	37	2022-11-29 Stem cell transplantation, anti-rejection by Rituximab and Basiliximab<br>2023-5-8 Diagnosis of Pulmonary infection	2023/5/11	FU.1
P4	Male	58	2023-7-1 Diagnosis of Herpes simplex encephalitis	2023/7/4	EG.5.2.2
P5	Male	82	2023/8/26 Diagnosis of Severe pneumonia	2023/8/27	EG.5.1.1
